# Prediction of response to neoadjuvant chemotherapy by MammaTyper® across breast cancer subtypes: A retrospective cross-sectional study

**DOI:** 10.1016/j.breast.2024.103753

**Published:** 2024-05-25

**Authors:** Francesco Schettini, Silvana Saracchini, Anna Bassini, Wally Marus, Serena Corsetti, Ilaria Specogna, Manuela Bertola, Elvia Micheli, Ralph M. Wirtz, Mark Laible, Uğur Şahin, Carla Strina, Manuela Milani, Sergio Aguggini, Richard Tancredi, Elena Fiorio, Sandro Sulfaro, Daniele Generali

**Affiliations:** a Translational Genomics and Targeted Therapies in Solid Tumors Group, August Pi I Sunyer Biomedical Research Institute (IDIBAPS), Barcelona, Spain; b Department of Medical Oncology, Hospital Clinic of Barcelona, Barcelona, Spain; c Faculty of Medicine and Health Sciences, Universitat de Barcelona, Barcelona, Spain; d IRCCS CRO di Aviano, Aviano, Italy; e Azienda per l’Assistenza Sanitaria 5 Friuli Occidentale, “Santa Maria degli Angeli” Hospital, Pordenone, Italy; f STRATIFYER Molecular Pathology GmbH, Cologne, Germany; g BioNTech SE, Mainz, Germany; h Multidisciplinary Unit of Breast Pathology and Translational Research, Cremona Hospital, Cremona, Italy; i Section of Oncology, Department of Medicine, University of Verona School of Medicine and Verona University Hospital Trust, 37134 Verona, Italy; j Department of Medical, Surgical and Health Sciences, University of Trieste, Trieste, Italy

**Keywords:** MammaTyper, Hormone receptor, Breast cancer, Subtypes, pCR, Neoadjuvant

## Abstract

**Background:**

Neoadjuvant chemotherapy (NACT) is widely used in the treatment of triple-negative and HER2-positive breast cancer (BC), but its use in estrogen receptor (ER) and/or progesterone receptor (PR) positive/HER2-negative BC is questioned because of the low pathologic complete response (pCR) rates. This retrospective study assessed the mRNA-based MammaTyper® assay's capability of predicting pCR with NACT, and ER, PR, Ki67, and HER2 status at immunohistochemical (IHC) through transcriptomics.

**Methods:**

Diagnostic biopsies from 76 BC patients treated at the Cremona Hospital between 2012-2018 were analyzed. Relative mRNA expression levels of *ERBB2, ESR1, PGR,* and *MKI67* were measured using the MammaTyper® kit and integrated into a pCR score. Predicting capability of pCR and standard IHC biomarkers could be assessed with ROC curves in 75 and 76 patients, respectively.

**Results:**

Overall, 68.0% patients obtained a MammaTyper® high-score and 32.0% a MammaTyper® low-score. Among high-score patients, 62.7% achieved pCR, compared to 16.7% in the low-score group (p = 0.0003). The binary MammaTyper® score showed good prediction of pCR in the overall cohort (area under curve [AUC] = 0.756) and in HR+/HER2-negative cases (AUC = 0.774). In cases with residual disease, the continuous MammaTyper® score correlated moderately with residual tumor size and decrease in tumor size. MammaTyper® showed substantial agreement with IHC for *ESR1*/ER and *ERBB2*/HER2, and moderate agreement for *PGR*/PR and *MKI67*/Ki67.

**Conclusion:**

Overall, MammaTyper® pCR score may serve as a standardized tool for predicting NACT response in HR+/HER2-negative BC, potentially guiding treatment strategies. Additionally, it could provide a more standardized and reproducible assessment of ER, PR, HER2, and Ki67 status.

## Highlights

• 76 neoadjuvant chemotherapy-treated breast cancer (BC) patients at the Cremona Hospital between 2012 and 2018 were included.
• MammaTyper classified 68.0 % as high-score and 32.0 % as low-score. In the first, 62.7 % achieved pCR vs. 16.7 % in the latter.
• MammaTyper score effectively predicted pCR in all patients (AUC = 0.76) and HR+/HER2- BC (AUC = 0.77), not in HER2+ and TN.
• The agreement between mRNA levels and IHC *ESR1*/ER and *ERBB2*/HER2 was substantial, and moderate for *PGR*/PR and *MKI67*/Ki67.
• MammaTyper continuous pCR score correlated moderately with residual tumor size and percentage decrease in tumor size.

## 1 Introduction

Neoadjuvant chemotherapy (NACT) is an increasingly common therapeutic approach for localized breast cancer (BC), especially in HER2-positive(+) and triple negative (TN) disease, since the achievement of pathological complete response (pCR) is strongly associated with improved long-term outcomes [1,2], and the presence of residual disease (RD) allows for further adjuvant treatments [[3], [4], [5]]. Moreover, NACT can facilitate breast conservation by tumor downstaging and convert initially inoperable breast tumors to operable [6]. In hormone receptor-positive (HR+)/HER2-negative BC, pCR rates are usually lower than TN and HER2+ and no post-neoadjuvant post-surgical therapeutic strategies have been implemented so far [1,2]. Thus, effective predictors of pCR are required to correctly identify patients that might benefit most from a neoadjuvant approach [6,7].

At present, menopausal status, primary tumor dimension (T), axillary nodal status (N), estrogen receptor (ER), progesterone receptor (PR), Ki67 and HER2 at diagnosis are the parameters that broadly guide therapeutic choices in early BC [8]. The latest four biomarkers are usually detected at the protein level with immunohistochemistry (IHC), but their reproducibility is suboptimal [9]. In this perspective, MammaTyper® is a CE marked *in-vitro* diagnostic device (IVD) which detects the levels of mRNA transcripts of the genes *ERBB2*, *ESR1*, *PGR*, and *MKI67* (i.e. HER2, ER, PR and Ki67 genes, respectively) on the basis of reverse transcription-quantitative real-time polymerase chain reaction (RT-qPCR) in a quick, standardized and observer-independent fashion [10]. The test is used for molecular subtyping of BC tissue into Luminal A-like, Luminal B-like/HER2-negative, Luminal B-like/HER2+, HER2+ (non-luminal) and TN tumors, as defined according to the St. Gallen 2013 Consensus [11]. The MammaTyper® assay can be used to predict the probability of pCR after NACT by integrating the assessment of *ERBB2*, *ESR1*, *PGR* and *MKI6*7 mRNA into a standardized prediction model, i.e. the MammaTyper® pCR score, which was initially validated in a prospectively planned analysis using archived samples from two clinical trials (TECHNO & PREPARE) [12].

In this retrospective study, we tested the MammaTyper® score as a predictor of pCR and MammaTyper® genomic biomarkers’ concordance with standard IHC parameters for BC subtyping in a real-world cohort from a single center Institution. Additionally, Jaruss JS et al. showed that a combination of pretreatment clinical stage (CS), baseline estrogen receptor (ER) status, tumor grade (G), and post-treatment pathologic stage (PS) into a score called CPS + EG, allowed for better determination of BC–specific survival than CS or PS alone in the neoadjuvant scenario [13]. Hence, we hereby explored the potential association of the MammaTyper® pCR score with CPS + EG.

## 2 Materials and methods

### 2.1 Patients and variables

We retrospectively identified archived formalin-fixed paraffin-embedded (FFPE) diagnostic biopsies from patients with unifocal early-stage BC treated with NACT at the Cremona Hospital between 2012 and 2018. Tumor cell content of ≥20 % was required for inclusion and confirmed on a hematoxylin & eosin (H&E) stained slide. Clinicopathological data were retrieved from patients' charts (list in Supplementary materials). The achievement of a pCR was assessed locally by dedicated breast pathologists for clinical practice purposes. A pCR was obtained when a residual tumor was classified as ypT0/Tis and ypN0 (absence of invasive cancer in breast and axilla) according to the AJCC's TNM staging system [14].

Immunohistochemical (IHC) tumor subtypes were classified according to the following cut-offs: ER/PR <1 %/≥1 %, Ki67 < 20 %/≥20 %, HER2+ if 3+ or 2+ and *in-situ* hybridization (ISH) positive. The CPS + EG score was calculated according to the methodology reported by Mittendorf EA et al. [15].

### 2.2 Study objectives: primary objective

To validate in a clinical practice cohort the MammaTyper® pCR score for prediction of response to NACT using the predefined cut-off (<42/≥42) to separate tumors with a low response rate from tumors with a high response rate, based on the initial core-needle biopsy taken prior to neoadjuvant treatment.

### 2.3 Study objectives: secondary objectives

• To assess the pCR predicting capability of the MammaTyper® score in the following subgroups: cT1-2 tumors, *ESR1-* or *PGR*-positive/*ERRB2*-negative, *ESR1-* or *PGR*-positive/*ERBB2*-positive, *ESR1-* and PGR-negative/*ERBB2*-negative, *ESR1*- and *PGR*-negative/*ERBB2*-positive, *ESR1-* or *PGR*-positive irrespective of *ERBB2*, *ERBB2*-positive irrespective of *ESR1* and *PGR,* as defined by MammaTyper® and, in case of discrepancy, as defined locally by IHC/ISH, as well.
• To assess pCR rates according to the MammaTyper® score class in the following subgroups: IHC-defined HR+/HER2-negative, HR+/HER2+, HR-negative/HER2+ and TN breast tumors, as well as in molecularly *ESR1* and/or *PGR*+/*ERRB2*-negative, *ESR1* and/or *PGR*+/*ERBB2*+, *ESR1* and *PGR*-negative/*ERBB2-*negative, *ESR1* and *PGR*-negative/*ERBB2+* and compare potential differences between the respective IHC and molecularly-defined subgroups.
• To assess the agreement between RT-qPCR-based and IHC/ISH-based determination of the markers *ERBB2*/HER2, *ESR1*/ER, *PGR*/PR and *MKI67*/Ki67 using the standard MammaTyper® cut-offs.
• To assess the association with and prediction of pCR of the continuous *ERRB2* mRNA levels in *ESR1* or *PGR* positive cases and *ESR1* or *PGR* negative cases from patients treated with anti-HER2 therapy.
• To assess the association with and prediction of pCR of the continuous *MKI6*7 mRNA levels in molecularly-defined TN cases.
• To assess the correlation of continuous MammaTyper® score values with tumor size in patients not achieving pCR.
• To assess the correlation and association of the MammaTyper® score, as continuous and dichotomic variable, with the CPS + EG prognostic score.

### 2.4 Laboratory methods

ER, PR, HER2 and Ki67 were assessed locally according to the respective ASCO/CAP guidelines [[16], [17], [18]]. Pre-treatment tumor samples were analyzed with MammaTyper® at BioNTech Diagnostics (Mainz, Germany), as elsewhere described [19]. The dedicated personnel at BioNTech Diagnostics was blinded to diagnostic IHC and clinical outcomes until the final analysis was finished. RNA extraction and MammaTyper® technical details are described in Supplementary materials. The MammaTyper® pCR score, as assessed by a proprietary algorithm and expressed by a number between 1 and 100 (Supplementary materials), was established on and has been applied to archived diagnostic biopsies from of all BC subtypes. In the MammaTyper TECHNO/PREPARE neoadjuvant trial, the MammaTyper® score stratified patients according to their response into two response zones: low-response (zone 1) and high-response (zone 2); these zones were divided by a single cut-off (<42/≥42) [12].

### 2.5 Statistical analysis

The prediction capability of the binary MammaTyper® pCR score was analyzed with regard to analytical accuracy (sensitivity, specificity, positive predictive value [PPV], negative predictive value [NPV], receiver operator characteristic [ROC] analysis). Association of the continuous MammaTyper® score with measures of partial response was analyzed in incomplete-responders by Spearman correlation. Agreement of IHC/ISH and mRNA-based single marker assessments was characterized using the measures positive percent agreement (PPA), negative percent agreement (NPA), overall percent agreement (OPA) and Kappa statistic. For the mRNA measurements predefined cut-offs (RU = 0) for positivity were used. Differences between groups of interest were assessed with χ^2^ test and Mann-Whitney *U* test, where appropriate. Association of *ERBB2* and *MKI67* levels with pCR were assessed with logistic regression. Linear and logistic regression were used to explore the association between the MammaTyper® pCR score and CPS + EG score as continuous or dichotomic (0–2 vs. 3–6) variable, respectively. Statistical significance was set at p ≤ 0.05. All analyses were conducted with R vers. 3.6.1 [20] for MacOSX and SAS/STAT® software vers. 9.4 (SAS Institute Inc., Cary, NC, USA) for Microsoft® Windows®.

## 3 Results

### 3.1 Population characteristics and MammaTyper® pCR score according to pathologic response

**Fig. 1:**
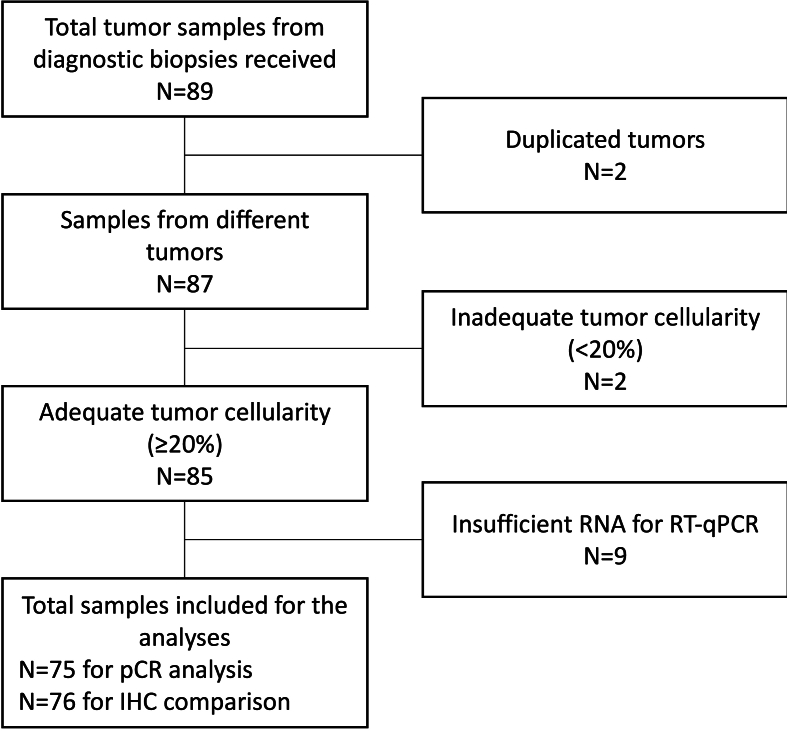
REMARK flow-chart. IHC: immunohistochemistry; pCR: pathologic complete response; RT-qPCR: real-time quantitative polymerase chain reaction.

A total of 76 patients with available FFPE tumor sample from pre-therapeutic biopsies obtained between 2012 and 2018 and treated with standard NACT, with or without anti-HER2 agents, at the Cremona Hospital were retrospectively included in this study (Fig. 1).

Thirty-nine patients (52.0 %) showed RD in the surgical sample after NACT and surgery, as compared to 36 (48.0 %) that achieved a pCR. One patient achieved a radiologic complete response at PET/TAC but refused to undergo breast surgery, hence was excluded from pCR analysis. Clinicopathologic features at diagnosis of the entire population are reported in Table 1. The extension of the residual tumors according to TNM are reported in Supplementary Table S1. When separating patients in a cohort achieving pCR and a cohort with RD, no differences at baseline were observed in terms of age (p = 0.4503), menopausal status (p = 0.4435), G (p = 0.3350), N (p = 0.2497), HER2 status by IHC (p = 0.0894), *ERBB2* status by MammaTyper® (p = 0.1371), Ki67 status by IHC (p = 0.3440) and *MKI67* status by MammaTyper® (p = 0.9186). Conversely, when comparing patients with RD to patients with pCR, a higher prevalence of ER-positive (+) (84.6 % vs. 38.9 %, p < 0.0001) and PR+ (71.8 % vs. 33.3 %, p = 0.0004) tumors as detected *via* IHC, as well as *ESR1+* (84.6 % vs. 33.3 %, p < 0.0001) and *PGR+* (56.4 % vs. 33.3 %, p = 0.0449) tumors as detected *via* RT-qPCR, was observed. Patients in the pCR cohort had more frequently a T1-2 tumor at diagnosis (88.9 % vs. 51.3 %) than patients in the RD cohort (p = 0.0004), where tumors were more frequently T3-4 (25.3 % vs. 5.3 %).

**Table 1:** Characteristics of the population included in the primary analysis at the time of diagnosis.

Clinicopathological characteristics including pre-therapeutic biopsies	Overall		pCR		non-pCR		*P*
	N	%	N	%	N	%	
	75	100.0	36	48.0	39	52.0	
**Age**							
<50 yr	30	40.0	16	44.4	14	35.9	0.4503
≥50 yr	45	60.0	20	55.6	25	64.1	
**Menopausal status**							
Premenopausal	32	42.7	17	47.2	15	38.5	0.4435
Postmenopausal	43	57.3	19	52.8	24	61.5	
**Tumor grade**							
1	0	0.0	0	0.0	0	0.0	0.3350
2	1	1.3	0	0.0	1	2.6	
3	74	98.7	36	48.0	38	50.7	
**Tumor size (cT)**							
cT1	16	21.3	12	33.3	4	10.3	*0.0004*
cT2	36	48.0	20	55.6	16	41.0	
cT3	9	12.0	4	5.3	5	6.7	
cT4	14	18.7	0	0.0	14	18.7	
**Axillary nodal status (cN)**							
cN0	24	32.9	14	38.9	10	25.6	0.2497
cN1	27	37.0	10	27.8	17	43.6	
cN2	8	11.0	3	4.1	5	6.8	
cN3	14	19.2	9	12.3	5	6.8	
*Missing*	2	2.7	0	0.0	2	2.7	
**ER (IHC)**							
Negative (<1 %)	28	37.3	22	61.1	6	15.4	*<0.0001*
Positive (≥1 %)	47	62.7	14	38.9	33	84.6	
**PR (IHC)**							
Negative (<1 %)	35	46.7	24	66.7	11	28.2	*0.0009*
Positive (≥1 %)	40	53.3	12	33.3	28	71.8	
**HER2 (IHC/ISH)**							
Negative (0, 1+, 2+/ISH-neg.)	45	60.0	18	50.0	27	69.2	0.0894
Positive (3+, 2+/ISH-pos.)	30	40.0	18	50.0	12	30.8	
**Ki67 (IHC)**							
Low (<20 %)	4	5.3	1	2.8	3	7.7	0.3440
High (≥20 %)	71	94.7	35	97.2	36	92.3	
***ESR1* (mRNA)**							
Negative	30	40.0	24	66.7	6	15.4	*<0.0001*
Positive	45	60.0	12	33.3	33	84.6	
***PGR* (mRNA)**							
Negative	41	54.7	24	66.7	17	43.6	*0.0449*
Positive	34	45.3	12	33.3	22	56.4	
***ERBB2* (mRNA)**							
Negative	44	58.7	17	47.2	27	69.2	0.1371
Positive	27	36.0	17	47.2	10	25.6	
Equivocal	4	5.3	2	5.6	2	5.1	
***MKI67* (mRNA)**							
Low	6	8.0	3	8.3	3	7.7	0.9186
High	69	92.0	33	91.7	36	92.3	
**Systemic treatment**							
Anthracycline + taxane-based	70	93.3	33	91.7	37	94.9	0.5783
Taxane-based	5	6.7	3	8.3	2	5.1	
With anti-HER2	33	44.0	20	55.6	13	33.3	0.0528
Without anti-HER2	42	56.0	16	44.4	26	66.7	
With carboplatin	15	20.0	12	33.3	3	7.7	*0.0055*
Without carboplatin	60	80.0	24	66.7	36	92.3	
**Surgical treatment**							
Quadrantectomy + SLNB±ALND	28	37.3	15	41.7	13	33.3	*0.4560*
Mastectomy + SLNB±ALND	47	62.7	21	58.3	26	66.7	
MammaTyper® pCR score* continuous							
Median	54.5	–	67	–	41	–	*0.0001*
Min - max	11–100	–	20–90	–	11–100	–	
MammaTyper® pCR score* class							
Low	24	32.0	4	11.1	20	51.3	*0.0002*
High	51	68.0	32	88.9	19	48.7	

CR: complete response; RD: residual disease; IHC: immunohistochemistry; ISH: *in situ* hybridization; pos.: positive; neg.: negative; min: minimum; max: maximum; pCR: pathologic complete response; SLNB: sentinel lymph-node biopsy; ALND: axillary lymph-node dissection; c: clinical staging at baseline. *MammaTyper*®* pCR score, as calculated in diagnostic biopsies.

Almost all patients received a NACT based on anthracyclines and taxanes and the proportion of patients receiving also anti-HER2 agents did not differ between patients achieving pCR or with RD (p = 0.0528), consistently with the absence of a significantly different distribution of HER2+ cases. However, more patients in the RD cohort did not receive carboplatin (p = 0.0055), due to an imbalance in TN cases between the 2 cohorts, since 11/16 TN tumors achieved pCR. Finally, tumors in the pCR cohort had higher median MammaTyper® continuous score (67 vs. 41, p = 0.0001), which translated in a higher proportion of tumors falling in the MammaTyper® high-score group (88.9 % vs. 48.7 %, p = 0.0002).

### 3.2 MammaTyper® pCR score and prediction of pCR

**Fig. 2:**
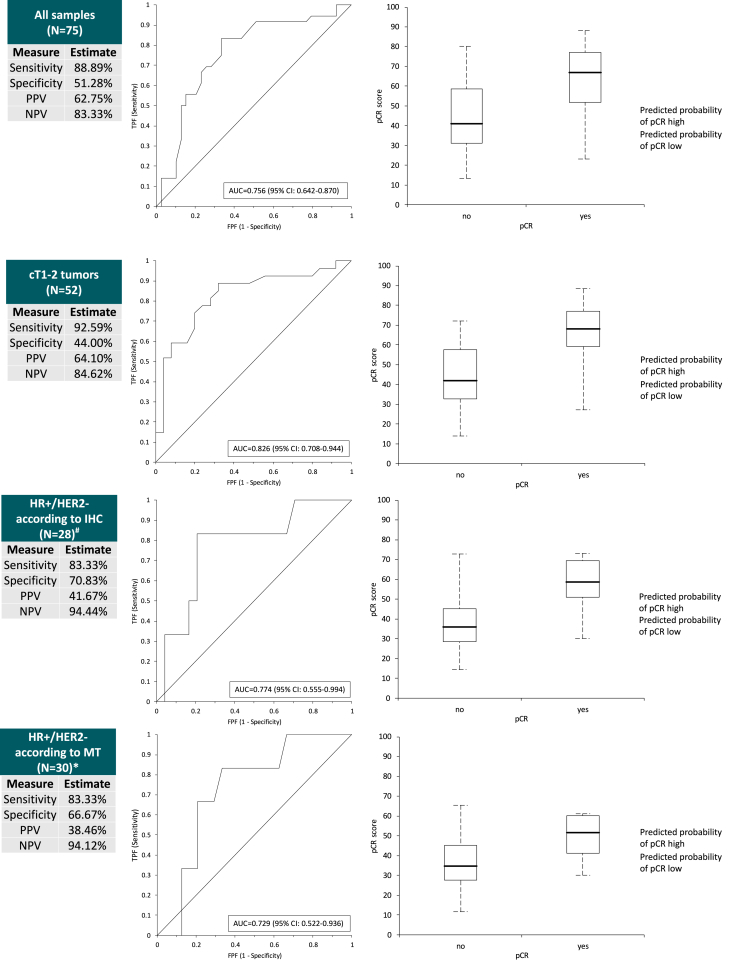
MammaTyper® pCR score prediction capability in the overall cohort and subgroups of interest. pCR: pathologic complete response; HR+: hormone receptor-positive; HER2-: HER2-negative; PPV: predictive positive value; NPV: negative positive value; AUC: area under curve; CI: confidence interval; IHC: immunohistochemistry; MT: MammaTyper®. *: 33 in principle, of which 2 excluded for *ERBB2* equivocal at MT and 1 excluded for not undergoing surgery; #: 29 in principle, of which 1 excluded for not undergoing surgery.

MammaTyper® continuous pCR score was significantly associated to the achievement of pCR (OR: 1.05, 95%CI: 1.02–1.08, p = 0.0004) after neoadjuvant treatment. Similarly, a high MammaTyper® score class was significantly associated to pCR, when compared to low MammaTyper® score class (OR: 8.42, 95%CI: 2.50–28.36, p = 0.0006). In fact, within the 51 high-score cases, 62.7 % achieved pCR after NACT, while in the low-score cases, only 16.7 % did (p = 0.0003). When we assessed the prediction capability of this score, we observed an AUC of 0.76 (95%CI: 0.64–0.87), with a sensitivity of 88.9 %, a specificity of 51.3 %, a PPV of 62.8 % and a NPV of 83.3 % (Fig. 2).

A similar predictive capability was observed when restricting the population to cT1-2/cN0-2 tumors (i.e. 20 HR+/HER2-negative, 12 HR+/HER2+, 7 HR-negative/HER2+ and 13 TN at IHC), or when only considering HR+/HER2-negative disease as *per* conventional IHC detection or according to MammaTyper® classification (Fig. 2).

However, the score lost significance in a multivariate model accounting for T, HER2, HR, both when considered as continuous (p = 0.204) or categorical (p = 0.096). Therefore, we evaluated its association to pCR in HR+ tumors, in HR-negative/HER2+ and TN separately. In HR+ tumors the MammaTyper® pCR score was significantly associated to pCR, both as continuous variable (OR: 1.04, 95%CI: 1.00–1.09, p = 0.0360) or class (OR: 4.23, 95%CI: 1.11–16.17, p = 0.0350). The score was associated with independence to HER2 status (positive vs. negative) and T (T2-3 vs. T1) when considered as continuous variable (adjusted OR: 1.05, 95%CI: 1.00–1.09, p = 0.0469), although the significance was lost when categorizing patients according to the predefined cut-off of 42 (p = 0.1195). Within HR-negative/HER2+ disease, the MammaTyper® pCR score was not associated to pCR (p = 0.2991) but all patients had been classified as MammaTyper® score high. Similarly, in the TN cohort, MammaTyper® pCR continuous score was not associated to pCR (p = 0.9254), but all cases had been classified as MammaTyper® score high.

**Fig. 3:**
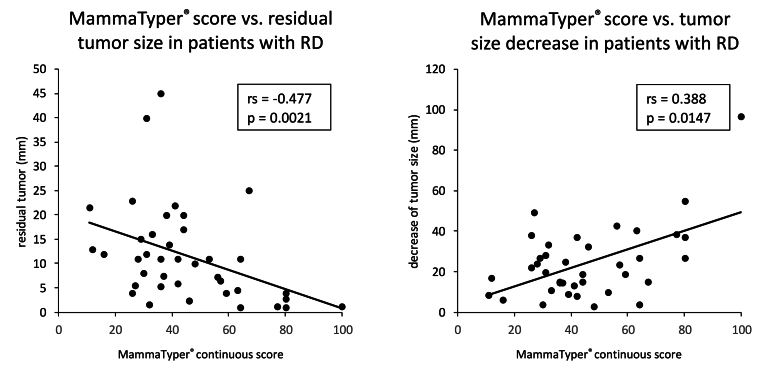
Correlation between MammaTyper® pCR score and residual tumor dimension/tumor size decrease in patients not achieving pCR. rs: Spearman's rho; RD: residual disease; mm: millimeters.

Finally, within the RD cohort, we observed a significantly moderate indirect correlation between the MammaTyper® continuous score and the dimension in mm of residual tumor (Spearman's rho: -0.48, p = 0.0021), as well as a direct weak correlation with the decrease in tumor size (Spearman's rho: 0.39, p = 0.0147), meaning the higher the score, the higher the tumor reduction and the lower the residual tumor dimension (Fig. 3).

### 3.3 pCR rates according MammaTyper® pCR score class and breast cancer subtype

**Fig. 4:**
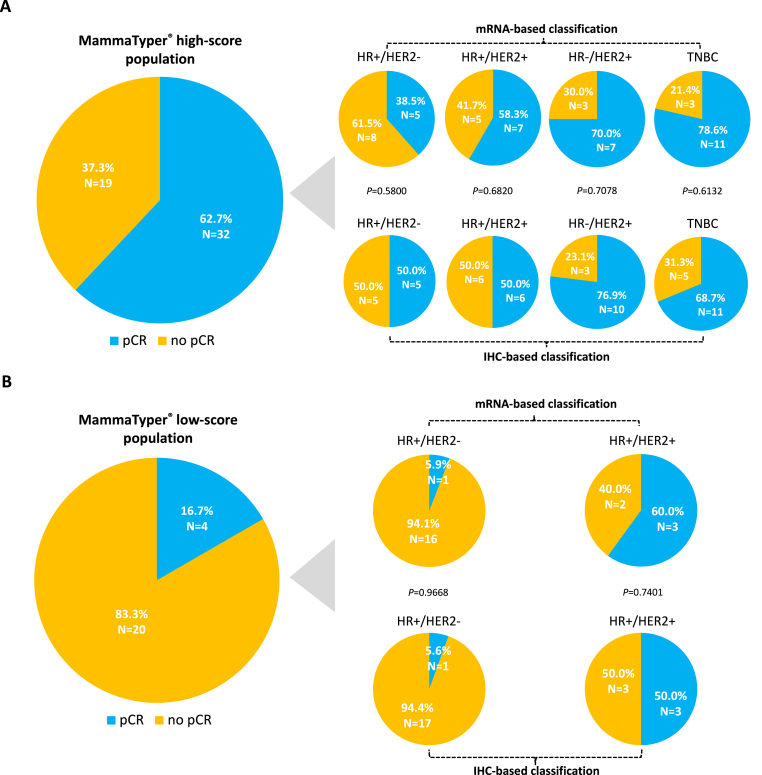
pCR rates according to MammaTyper® pCR score class and surrogate subtype classification. pCR: pathologic complete response; HR: hormone receptor; TNBC: triple negative breast cancer; +: positive; −: negative; IHC: immunohistochemical. In the IHC-based classification, in case of HER2 2+ IHC score, hybridization *in situ* technique was used to assess *ERBB2* gene amplification status. Tumors with an *ERBB2* equivocal result at MammaTyper*®* were excluded from the molecular classification; two cases pertained to the MammaTyper*®* low-score group and two pertained to the high-score group, resulting in a total number of 22 and 49 patients available for the analysis, respectively, instead of 24 and 51 by IHC. One patient was excluded from the pCR analysis for refusing surgery. This case pertained to the MammaTyper® pCR low-score cohort. Within the same MammaTyper*®* pCR score group, each IHC-based subtype was compared to the corresponding mRNA-based subtype to assess potential differences in the proportion of patients achieving or not a pCR. *P* values are referred to χ^2^ tests conducted between each subtype couple (IHC-based classification vs. mRNA-based classification by MammaTyper*®*) within the same MammaTyper*®* score class (high or low).

When comparing pCR rates depending on MammaTyper® score class and BC subtype, we observed that cases classified as MammaTyper® score high had a significantly higher pCR rate than those classified as MammaTyper® score low only within the HR+/HER2-negative subtype, both when the subtype was defined according to standard IHC (pCR rate of 50.0 % vs. 5.6 %, respectively, p = 0.0060) or MammaTyper® (pCR rate of 38.5 % vs. 5.9 %, respectively, p = 0.0271). In the case of HR+/HER2+ disease, similar when not identical pCR rates were observed between the MammaTyper® score high and low cohorts, both with IHC-based and mRNA-based subtype classification. Furthermore, as previously mentioned, within HR-negative/HER2+ and TN cases, tumors were only classified as MammaTyper® score high. When we compared the pCR rates within the same MammaTyper® score class between each IHC-detected subtype and the respective molecularly-defined subtype, no significant difference was observed (Fig. 4).

### 3.4 Association of the MammaTyper® pCR score with the CPS + EG score

The distribution of CPS + EG scores in the overall population and according to pathologic status after surgery is reported in Supplementary Table S2. At the univariate linear and logistic regression, the continuous and dichotomic (high vs. low) MammaTyper® pCR score was not significantly associated with CPS + EG. However, in the subset of HR+/HER2-negative disease, a significant inverse correlation between CPS + EG score as continuous variable and the MammaTyper® continuous score (Pearson's r: -0.469, p = 0.009; coeff. B: -0.029, adjusted R^2^: 0.18, p = 0.017) and dichotomic score (Pearson's r: -0.373, p = 0.030; coeff. B: -0.869, adjusted R^2^: 0.10, p = 0.060) was observed, though only moderate. Similarly, the MammaTyper® continuous (OR: 0.91, p = 0.035) and dichotomic score (OR: 0.09, p = 0.037) were significantly less associated to a CPS + EG of 3–6 than 0–2 (Supplementary results).

### 3.5 ERBB2 and MKI67 levels’ associations with pCR in molecular subgroups and MammaTyper® prediction of molecular subtypes

Within HER2+ tumors as *per* MammaTyper® classification, treated with anti-HER2 agents and NACT, we explored *ERBB2* mRNA levels’ association with pCR. No significant association was observed in the overall cohort (p = 0.396), in HR+/HER2+ (p = 0.566) and HR-negative/HER2+ (p = 0.488) disease, respectively. Thus, prediction capability was not further explored. Similarly, for molecularly-defined TN tumors, *MKI67* levels were not found to be associated to pCR (p = 0.348), hence prediction capability was not further explored.

**Fig. 5:**
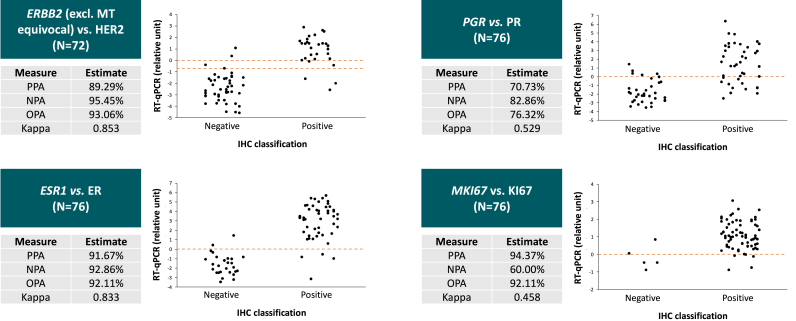
Predicting capability of IHC biomarkers with transcriptomics. ER: estrogen receptor; PR: progesterone receptor; PPA: positive percent agreement; NPA: negative percent agreement; OPA: overall percent agreement; Kappa: Cohen's Kappa; IHC: immunohistochemical; RT-qPCR: reverse transcriptase quantitative polymerase chain reaction; MT: MammaTyper®.

When we tested MammaTyper® capability of predicting ER, PR, Ki67 and HER2 status (positive and negative in all cases), we observed an almost perfect agreement for *ESR1*/ER (Kappa: 0.83, PPA: 91.7 %, NPA: 92.9 %, OPA 92.1 %) and *ERBB2*/HER2 (Kappa: 0.85, PPA: 89.3 %, NPA: 95.5 %, OPA 93.1 %). Regarding *ERBB2*, a total of 4 equivocal cases at MammaTyper® were excluded from the present analysis. A statistically significant moderate agreement was observed between *PGR*/PR (Kappa: 0.53) and *MKI67*/Ki67 (Kappa: 0.46) (Fig. 5).

## 4 Discussion

In this retrospective single-center study, we assessed for the first time in a clinical practice cohort the capability of the standardized MammaTyper® RT-qPCR assay to predict ER, PR, HER2 and Ki67 status, through the detection of their respective mRNA levels, and the association of the MammaTyper® pCR score with response to NACT. We observed that the assay may predict efficiently BC IHC surrogate subtypes. Additionally, MammaTyper® pCR score may serve as a standardized tool to predict response to NACT based on a pre-treatment biopsy, especially in luminal-like disease.

Nowadays, for HER2+ and TN breast tumors beyond 1.5–2 cm, a neoadjuvant approach is the therapeutic standard to grant patients access to additional adjuvant treatments if pCR is not achieved [8,21]. In this context, MammaTyper® was not able to add pCR prediction beyond standard HR and HER2 IHC status, since all cases were assigned to the MammaTyper® high-score group. To note, the HER2DX® genomic assay, integrating also T and N, already proved to efficiently predict pCR, prognosis and sensitivity to anti-HER2 agent-based regimens beyond classical IHC features [22,23]. In TN tumors, there is still no multiparametric test available, however tumor-infiltrating lymphocytes, from one side, or the integration of the immunologic IGG signature with T and N in a multiparametric prognostic score, might identify patients with a very good prognosis [[24], [25], [26]], who might be candidates for de-/escalated therapeutic approaches, either before surgery, or subsequently, so to adapt post-surgical treatments not only on the presence of pCR/RD but also on additional features. Also in TN, among 907 clinical-genomic biomarkers assessed in the CALGB40603 trial, several of them were associated with pCR, event-free survival or both [27]. Given the previous, we believe there is little room for MammaTyper® in the context of HER2+ and TN early-stage disease for pCR prediction.

MammaTyper® successfully identified a group of patients more prone to achieve pCR within HR+/HER2-negative tumors. No differential treatments are currently provided to patients with pCR or RD in this BC subset. Notably, although the neoadjuvant therapeutic approach has been demonstrated to be substantially equivalent to the adjuvant approach in terms of impact on long-term outcomes [28], several patients feel uncomfortable at the time of delaying surgery when the tumor is operable and there are no specific surgical or post-surgical needs, with surgery acting as a relevant factor of distress and anxiety reduction [29,30]. However, the neoadjuvant approach, also for these patients, might be useful to predict treatments activity *in vivo*. Furthermore, it has been demonstrated that a significant reduction in tumor burden from diagnosis to surgery after neoadjuvant therapy, is able to provide a significant prognostic information *per se*, beyond the mere achievement of pCR, as exemplified by calculating the residual cancer burden (RCB) score [1,2]. In this perspective, it is worth nothing that MammaTyper® score as continuous variable was significantly directly associated to a tumor reduction in mm and inversely associated to post-neoadjuvant primary tumor dimension in mm. Also notably, we observed that within HR+/HER2-negative disease, the MammaTyper score® was inversely, though only moderately, associated to the CPS + EG score, which is a powerful prognostic score, validated in HR+/HER2-negative disease undergoing NACT, and inversely associated to long-term outcomes [13,15,31]. Finally, the neoadjuvant approach is a perfect setting for drug and biomarker development and several trials are testing the feasibility of potentially valuable adjuvant treatments in a scenario where standard treatment can be still administered after surgery without impairing patients outcomes [6,32]. For all these considerations, we believe that MammaTyper® might play a role in selecting patients with HR+/HER2-negative BC at higher probability of obtaining a significant tumor shrinkage with a neoadjuvant approach, inside or outside of a clinical trial, given the positive prognostic implications. Furthermore, for patients with inoperable luminal tumors and low predicted probability of pCR according to MammaTyper®, neoadjuvant endocrine therapy (NET), alone or combined with other agents like CDK4/6-inhibitors or alternative targeted agents, inside or outside of clinical trials, may represent an alternative strategy to pursue tumor downstaging.

Importantly, some of the most important parameters that we use in our clinical practice for therapeutic decision-making worldwide, namely ER, PR, HER2 and Ki67, are subject to a certain degree of heterogeneity in their assessment. One first reason is methodological and based on the plurality of antibodies against ER and PR, or HER2, the concordance of which is far from being perfect [9]. Regarding HR+/HER2-negative disease, the capability of correctly identifying luminal-like tumors from TN is crucial, since up to a 13 % of HR + tumors are characterized by low levels of ER and PR (IHC below 10 %) [33,34]. These tumors are molecularly Basal-like in 48–62 % cases, and act like TN disease. However, a proportion of HR-low tumors is not Basal-like and behave like luminal disease [[33], [34], [35], [36], [37]]. Also, within luminal tumors a Luminal A and Luminal B subtypes exist, with different endocrine sensitiveness, proliferation activity and chemosensitivity [36,38]. At present, if molecular intrinsic subtyping cannot be performed, the levels of Ki67 are used to discriminate between Luminal A-like and Luminal B-like disease, according to the St. Gallen Consensus criteria [11]. Unfortunately, Ki67 precise assessment is challenging and there is significant inter-laboratory disagreement [[39], [40], [41], [42]]. Many therapeutic decision are currently based on Ki67 detection, like whether to administer adjuvant CT if genomic tests are not available, or after standard or short-term NET [8,43,44], and whether to administer or not adjuvant abemaciclib or ribociclib in some stage II and III cases [45,46]. Finally, HER2 assessment by IHC is prone to a certain disagreement among different pathologists, especially with regard to the new HER2-low category [47,48]. In all of these cases of potential uncertainty, MammaTyper®, being a relatively cheap, standardized, reproducible and operator-independent assay [10,19], might help in assessing key pathology biomarkers to guide therapeutic choices, especially in contexts where BC expert pathologists are not available or when the access to genomic assays for prognostication and chemotherapy prescription are not feasible. Noteworthy, the test has the potential to be integrated into the local laboratory setup because it supports analysis on widely accessible qPCR platforms using total RNA extracted from clinical routine FFPE BC samples [10]. Nevertheless, it should be kept in mind that the latest guidelines on early stage breast cancer, including the ones from the American Society of Clinical Oncology (ASCO), European Society for Medical Oncology (ESMO) and the German Gynecological Oncology Group (AGO) still endorse ER/PR/HER2/Ki67 assessment *via* IHC/ISH [16,[49], [50], [51]].

This study is not exempt from limitations, the more obvious relying in its retrospective nature; the low number of cases where the test could be applied, even more when subdividing the cohort in surrogate subtypes, also limited the possibility to efficiently test the associations of the MammaTyper® score with surgical outcomes, especially in the multivariable models. However, results were coherent with its initial validation study [12]. Moreover, it is the first time that MammaTyper® performance as predictor of pCR and predictor of ER, PR, HER2 and Ki67 status was tested in a clinical practice scenario, since a previous validation was conducted retrospectively in cohorts obtained from the phase II TECHNO and the phase III PREPARE clinical trials [12]. This assay might be useful to reduce the uncertainty in the identification of surrogate BC subtypes, especially when expert pathologists are not available or when genomic testing for CT prescription are not affordable. Regarding MammaTyper® pCR predictor, while its clinical utility is questionable in HER2+ and TN disease, it might help identifying luminal-like tumors that might be candidate to de-/escalated therapeutic strategies, either in the clinical practice or research scenario. Overall, MammaTyper® predicting capabilities should be ultimately tested in a wider cohort, with special focus on HR+/HER2-negative disease.

## Ethic statement

The study protocol was approved by the Local Ethics Committee Val Padana (IRB n.32,219) on December 21, 2018. According to Italian law, all patients provided written informed consent before participating the study, except for those who were untraceable.

## Funding

Molecular analyses and statistical support were provided by BioNTech. Mednote, spin-off of the University of Trieste, also provided funding support.

## Data availability

Anonymized data are available upon reasonable request from the corresponding authors.

## CRediT authorship contribution statement

**Francesco Schettini:** Writing – review & editing, Writing – original draft, Methodology, Investigation, Formal analysis, Conceptualization. **Silvana Saracchini:** Writing – review & editing, Writing – original draft, Investigation, Data curation. **Anna Bassini:** Writing – review & editing, Writing – original draft, Investigation, Data curation. **Wally Marus:** Writing – review & editing, Writing – original draft, Investigation, Data curation. **Serena Corsetti:** Writing – review & editing, Writing – original draft, Investigation, Data curation. **Ilaria Specogna:** Writing – review & editing, Writing – original draft, Data curation, Conceptualization. **Manuela Bertola:** Writing – review & editing, Writing – original draft, Investigation, Data curation. **Elvia Micheli:** Writing – review & editing, Writing – original draft, Investigation, Data curation. **Ralph M. Wirtz:** Writing – review & editing, Writing – original draft, Methodology, Investigation, Formal analysis. **Mark Laible:** Writing – review & editing, Writing – original draft, Methodology, Investigation, Formal analysis. **Uğur Şahin:** Writing – review & editing, Writing – original draft, Methodology, Investigation, Formal analysis. **Carla Strina:** Writing – review & editing, Data curation. **Manuela Milani:** Writing – review & editing, Data curation. **Sergio Aguggini:** Writing – review & editing, Data curation. **Richard Tancredi:** Writing – review & editing, Data curation. **Elena Fiorio:** Writing – review & editing, Data curation. **Sandro Sulfaro:** Writing – review & editing, Writing – original draft, Investigation, Data curation. **Daniele Generali:** Writing – review & editing, Writing – original draft, Supervision, Project administration, Methodology, Investigation, Funding acquisition, Conceptualization.

## Declaration of competing interest

Francesco Schettini reports honoraria from Novartis, Gilead and Daiichy-Sankyo for educational events/materials and travel expenses from Novartis, Gilead and Daiichy-Sankyo. Daniele Generali declares personal fees for educational events by Novartis, Lilly, Pfizer, Daiichy-Sankyo, Roche; research funds from Astrazeneca, 10.13039/100004336Novartis and LILT. Uğur Şahin is CEO and Mark Laible is an employee of BioNTech SE. Ralph M Wirtz is an employee of STRATIFYER Molecular Pathology GmbH.

The other authors have nothing to declare.
