# Supplementary material for: Prediction of response to neoadjuvant chemotherapy by MammaTyper® across breast cancer subtypes: A retrospective cross-sectional study
Source: Breast. 2024 May 25;76:103753. doi: 10.1016/j.breast.2024.103753 (PMC11166895; doi:10.1016/j.breast.2024.103753)
Supplement: Multimedia component 1 [file mmc1.docx]

**Supplementary materials**

[**Supplementary methods** 2](#_Toc157508448)

[List of clinicopathological variables retrieved 2](#_Toc157508449)

[MammaTyper^®^ laboratory methods and pCR score definition 2](#_Toc157508450)

[References 3](#_Toc157508451)

[**Supplementary results** 5](#_Toc157508452)

[Association of the MammaTyper^®^ pCR score with the CPS+EG score 5](#_Toc157508453)

[**Supplementary tables** 6](#_Toc157508454)

[Supplementary table 1. Detailed pathologic status after neoadjuvant treatment and surgery 6](#_Toc157508455)

[Supplementary table 2. CPS+EG score distribution between patients achieving or not pCR 7](#_Toc157508456)

# **Supplementary methods**

## **List of clinicopathological variables retrieved**

Pre-treatment clinicopathological data were retrieved from patients’ charts, namely age, menopausal status, tumor size (T), axillary lymph-node involvement (N), pathology grade, Ki67, estrogen receptor (ER), progesterone receptor (PR), HER2 by immunohistochemistry and *in-situ* hybridization, systemic/surgical treatment details and surgical outcomes in terms of type of surgery, post-surgical (yp)T, ypN and pCR achievement. The presence of these variables was mandatory for patients inclusion, with the exception of age, menopausal status and Ki67. When available, post-surgical ER, PR, HER2 and Ki67 were collected, as well.

## **MammaTyper^®^ laboratory methods and pCR score definition**

Formalin-fixed paraffin-embedded (FFPE) tumor samples from diagnostic biopsies with sufficient tumor cell content (at least 20%) were subjected to extraction of total RNA (1x10µl whole surface tissue section) using the RNXtract® kit (BioNTech Diagnostics), as per manufacturer’s instructions. This kit has been validated by the manufacturer for RNA extraction for MammaTyper^®^. Relative mRNA expression levels of *ERBB2*, *ESR1*, *PGR* , *MKI67* and two reference genes, namely *B2M* and *CALM2* in FFPE tumor samples were determined by RT-qPCR using the CE-IVD MammaTyper^®^ kit on a CFX96^TM^ qPCR system (BioRad), in accordance with Instructions for Use 150528-90020 revision 3.0. The analytical outputs were normalized and quantitative single-marker results were given as 40^−ΔΔCq^ (quantification cycle) values on a continuous scale. The MammaTyper^®^ tool then provides the status of each gene as a binary category – positive or negative – based on clinically validated marker- and device-specific cut-off values. The gene expression data could be then integrated so as to assign individual samples to a molecular subtype of breast cancer[1], as elsewhere described[2]. The continuous expression values were integrated into a prediction score (pCR score) using a predefined algorithm and cut-off[3]. More specifically, Fasching P et al. carried out a logistic regression analysis to train a multivariable model for prediction of pCR according to *ESR1, PGR, MKI67* and *ERBB2* mRNA levels according to MammaTyper^®^ measurement in a discovery cohort of 462 FFPE tumor samples from patients with early stage breast cancer from the Erlangen Neoadjuvant Study Breast (ERNEST-B) [3,4]. An unscaled score (s_u_) was obtained, defined by the following equation: –6.394 + (0.099 * 40-ddCq *ERBB2*) – (0.279 * 40-ddCq ESR1) – (0.108 * 40-ddCq PGR) + (0.426 * 40-ddCq MKI67). The score was further rescaled to a range between 0 and 100 to allow easier interpretation in clinical routine. The values that defined the four quartiles of the MammaTyper® pCR-score in the discovery cohort were: Q1, < 27; Q2, ≥ 27 and < 42; Q3, ≥ 42 and < 69; and Q4, ≥ 69 [3]. The median MammaTyper^®^ pCR-score was 42, and this cut-off was therefore taken forward to demarcate ‘low’ and ‘high’ scores in a validation cohort. The validation cohort included all patients from the intention-to-treat (ITT) sets of TECHNO and PREPARE prospective trials for whom tumor material, taken as a core biopsy before the start of study treatment, was still available [3]. In both studies, patients were treated with neoadjuvant chemotherapy, with or without anti-HER2 therapy with trastuzumab [5,6].

## **References**

[1] Schettini F, Brasó-Maristany F, Kuderer NM, Prat A. A perspective on the development and lack of interchangeability of the breast cancer intrinsic subtypes. NPJ Breast Cancer 2022;8:85. https://doi.org/10.1038/s41523-022-00451-9.

[2] Laible M, Schlombs K, Kaiser K, Veltrup E, Herlein S, Lakis S, et al. Technical validation of an RT-qPCR in vitro diagnostic test system for the determination of breast cancer molecular subtypes by quantification of ERBB2, ESR1, PGR and MKI67 mRNA levels from formalin-fixed paraffin-embedded breast tumor specimens. BMC Cancer. 2016 Jul 7;16:398. doi: 10.1186/s12885-016-2476-x.

[3] Fasching PA, Laible M, Weber KE, Wirtz RM, Denkert C, Schlombs K, et al. Validation of the MammaTyper® pathological complete response (pCR)-score as a predictor for response after neoadjuvant chemotherapy (NACT) in patients with early breast cancer (BC). Annals of Oncology 2018;29:viii53. https://doi.org/10.1093/annonc/mdy269.166.

[4] Wunderle M, Gass P, Häberle L, Flesch VM, Rauh C, Bani MR, Hack CC, Schrauder MG, Jud SM, Emons J, Erber R, Ekici AB, Hoyer J, Vasileiou G, Kraus C, Reis A, Hartmann A, Lux MP, Beckmann MW, Fasching PA, Hein A. BRCA mutations and their influence on pathological complete response and prognosis in a clinical cohort of neoadjuvantly treated breast cancer patients. Breast Cancer Res Treat. 2018 Aug;171(1):85-94. doi: 10.1007/s10549-018-4797-8.

[5] Untch M, Fasching PA, Konecny GE, Hasmüller S, Lebeau A, Kreienberg R, Camara O, Müller V, du Bois A, Kühn T, Stickeler E, Harbeck N, Höss C, Kahlert S, Beck T, Fett W, Mehta KM, von Minckwitz G, Loibl S. Pathologic complete response after neoadjuvant chemotherapy plus trastuzumab predicts favorable survival in human epidermal growth factor receptor 2-overexpressing breast cancer: results from the TECHNO trial of the AGO and GBG study groups. J Clin Oncol. 2011 Sep 1;29(25):3351-7. doi: 10.1200/JCO.2010.31.4930.

[6] Untch M, Fasching PA, Konecny GE, von Koch F, Conrad U, Fett W, Kurzeder C, Lück HJ, Stickeler E, Urbaczyk H, Liedtke B, Salat C, Harbeck N, Müller V, Schmidt M, Hasmüller S, Lenhard M, Schuster T, Nekljudova V, Lebeau A, Loibl S, von Minckwitz G; Arbeitsgemeinschaft Gynäkologische Onkologie PREPARE investigators. PREPARE trial: a randomized phase III trial comparing preoperative, dose-dense, dose-intensified chemotherapy with epirubicin, paclitaxel and CMF versus a standard-dosed epirubicin/cyclophosphamide followed by paclitaxel ± darbepoetin alfa in primary breast cancer--results at the time of surgery. Ann Oncol. 2011 Sep;22(9):1988-1998. doi: 10.1093/annonc/mdq709.

#

# **Supplementary results**

## **Association of the MammaTyper^®^ pCR score with the CPS+EG score**

We calculated the CPS+EG score for the 75 patients included in the pCR analysis, as elsewhere described (Mittendorf EA et al. J Clin Oncol 2011; 29:1956-1962). The distribution of CPS+EG scores in the overall population and according to pathologic status after surgery is reported in **Supplementary table 2**. We then used univariate linear regression to assess the association between the MammaTyper^®^ pCR score as continuous or categorical (high or low) variable with CPS+EG as continuous variable and univariate logistic regression to assess the association between the MammaTyper^®^ score as continuous or categorical variable with CPS+EG as a dichotomic score (values from 0 to 2 vs. values from 3 to 6). The continuous MammaTyper^®^ pCR score was not significantly associated with CPS+EG, both as continuous variable (p=0.135) and categoric variable (p=0.719). The same was observed with MammaTyper^®^ dichotomic score (high vs. low) and its association with CPS+EG, both as continuous variable (p=0.709) and dichotomic variable (p=0.322). Posteriorly, we focused on the subset of hormone receptor-positive (HR+)/HER2-negative disease. A significant inverse correlation between the MammaTyper^®^ continuous score and the CPS+EG score as continuous variable was observed, although the correlation was only moderate (Pearson’s r: -0.469, p=0.009) and the MammaTyper^®^ score variance could only explain less than 20% of the variance of CPS+EG (coeff. B: -0.029, adjusted R^2^: 0.18, p=0.017). When the MammaTyper^®^ pCR score was considered as a dichotomous variable, patients with a high score showed a significant but moderate inverse association with CPS+EG (Pearson’s r: -0.373, p=0.030; coeff. B: -0.869, adjusted R^2^: 0.10, p=0.060). Furthermore, the MammaTyper^®^ continuous (odds ratio [OR]: 0.91, 95% confidence interval [CI]: 0.84 – 0.99, p=0.035) and dichotomic score (OR: 0.09, 95%CI: 0.01 – 0.87, p=0.037) were significantly less associated to a CPS+EG of 3-6 than 0-2.

# **Supplementary tables**

## **Supplementary table 1. Detailed pathologic status after neoadjuvant treatment and surgery**

| **DETAILED PATHOLOGIC STATUS AFTER NACT** | **RESIDUAL DISEASE COHORT** | |
| --- | --- | --- |
|  | **N** | **%** |
| **ypT status** |  |  |
| *ypT0/is* | 0 | 0.0 |
| *ypT1* | 31 | 79.5 |
| *ypT2* | 7 | 17.9 |
| *ypT3* | 0 | 0.0 |
| *ypT4* | 1 | 2.6 |
| *Overall* | 39 | 100.0 |
| **ypN status** |  |  |
| *ypN0* | 23 | 59.0 |
| *ypN1mic/ITC/1** | 12 | 30.8 |
| *ypN2* | 1 | 2.6 |
| *ypN3* | 3 | 7.7 |
| *Overall* | 39 | 100.0 |

**Legend.** NACT: neoadjuvant chemotherapy; ITC: isolated tumor cells. *no ITC were observed in the study cohort.

## **Supplementary table 2. CPS+EG score distribution between patients achieving or not pCR**

| **CPS+EG** | **Overall** | | **pCR Cohort** | | **non-pCR Cohort** | | ***P*** |
| --- | --- | --- | --- | --- | --- | --- | --- |
|  | **N** | **%** | **N** | **%** | **N** | **%** |  |
| *0* | 0 | 0.0 | 0 | 0.0 | 0 | 0.0 | 0.0819 |
| *1* | 16 | 21.9 | 7 | 19.4 | 9 | 24.3 |  |
| *2* | 22 | 30.1 | 15 | 41.7 | 7 | 18.9 |  |
| *3* | 17 | 23.3 | 5 | 13.9 | 12 | 32.4 |  |
| *4* | 16 | 21.9 | 9 | 25.0 | 7 | 18.9 |  |
| *5* | 2 | 2.7 | 0 | 0.0 | 2 | 5.4 |  |
| *6* | 0 | 0.0 | 0 | 0.0 | 0 | 0.0 |  |
| *Overall* | 73* | 100.0 | 36 | 100.0 | 37 | 100.0 |  |

**Legend.** pCR: pathologic complete response. CPS+EG score was calculated according to Mittendorf EA et al. J Clin Oncol 2011; *29:1956-1962.* *: 2/76 cases had unknown axillary nodal status at baseline and 1/76 did not undergo surgery, hence CPS+EG score was not assessable for these patients.
